# Tripping Elicits Earlier and Larger Deviations in Linear Head Acceleration Compared to Slipping

**DOI:** 10.1371/journal.pone.0165670

**Published:** 2016-11-01

**Authors:** Sara L. Arena, Julian L. Davis, J. Wallace Grant, Michael L. Madigan

**Affiliations:** 1 Department of Exercise Science, High Point University, High Point, North Carolina, United States of America; 2 Department of Engineering, University of Southern Indiana, Evansville, Indiana, United States of America; 3 Department of Engineering Science & Mechanics, Virginia Polytechnic Institute and State University, Blacksburg, Virginia, United States of America; 4 Department of Biomedical Engineering, Texas A&M University, College Station, Texas, United States of America; University of Florida, UNITED STATES

## Abstract

Slipping and tripping contribute to a large number of falls and fall-related injuries. While the vestibular system is known to contribute to balance and fall prevention, it is unclear whether it contributes to detecting slip or trip onset. Therefore, the purpose of this study was to investigate the effects of slipping and tripping on head acceleration during walking. This information would help determine whether individuals with vestibular dysfunction are likely to be at a greater risk of falls due to slipping or tripping, and would inform the potential development of assistive devices providing augmented sensory feedback for vestibular dysfunction. Twelve young men were exposed to an unexpected slip or trip. Head acceleration was measured and transformed to an approximate location of the vestibular system. Peak linear acceleration in anterior, posterior, rightward, leftward, superior, and inferior directions were compared between slipping, tripping, and walking. Compared to walking, peak accelerations were up to 4.68 m/s^2^ higher after slipping, and up to 10.64 m/s^2^ higher after tripping. Head acceleration first deviated from walking 100-150ms after slip onset and 0-50ms after trip onset. The temporal characteristics of head acceleration support a possible contribution of the vestibular system to detecting trip onset, but not slip onset. Head acceleration after slipping and tripping also appeared to be sufficiently large to contribute to the balance recovery response.

## Introduction

Falls continue to be a major source of morbidity and mortality. In the workplace, falls accounted for 24% of disabling injuries in 2012 [1]. Slipping causes an estimated 40–50% of fall-related workplace injuries [2], and tripping causing 23–32% of workplace falls [3–5]. Among older adults, falls were responsible for over 67% of non-fatal injuries and 45% of injury-related deaths among older adults in 2010 [6]. Slipping and tripping combined account for an estimated 46% of falls among older adults [7]. Slipping commonly occurs while walking when the foot slips forward at heel contact, and the head/trunk subsequently falls backward. Tripping typically occurs while walking when the swing foot is obstructed, and the head/trunk subsequently falls forward. In both cases, a balance recovery response is needed to avert a fall. The more quickly the slip or trip can be detected and the recovery response initiated, the more likely a fall can be averted.

Maintaining balance is dependent upon sensory feedback from the visual, proprioceptive, and vestibular systems. Of particular interest here is the vestibular system, which detects head orientation/linear acceleration and angular acceleration using the otolith organs (utricle and saccule) and semicircular canals, respectively [8]. Sensory information from the vestibular system is utilized for upper body control across all phases of the gait cycle [9], possibly to contribute to head stabilization [10]. Head stabilization is desirable to minimize the effect gait-related oscillations on visual and vestibular inputs [10–12]. Sensory information from the vestibular system is also utilized for lower body control during gait, and particularly during double support when both feet are on the ground in order to assist in the planning of subsequent steps [13]. Double support also allows for information from the proprioceptive system to be integrated with vestibular information to generate an internal representation of the body in space [14] and estimate if the movement of the body relative to the base of support results in the desired end position [9].

The contribution of the vestibular system to maintaining balance varies depending upon the characteristics of perturbations to balance that are experienced. At low perturbation amplitudes (such as deviations from equilibrium during quiet standing), the vestibular system primarily contributes to balance by resolving conflicting sensory information from other sensory systems [15]. As perturbation amplitude increases, there is an increasing reliance on the vestibular system to maintain balance [16]. The location where the perturbation is applied can also influence the contributions of the vestibular system. Horak et al. showed when perturbations are applied near the head and result in early head motion, the vestibular system is primarily responsible for triggering recovery responses [17]. In contrast, when perturbations are applied more distally (e.g. the feet by a translating support surface) and result in later head motion, the proprioceptive system is primarily responsible for triggering recovery responses. Although both slips and trips involve perturbations applied distally to a foot, the differences in these perturbations (impulsive load applied to swing foot when tripping, and lack of friction force when slipping) could result in different time delays before alterations in head acceleration are observed. As a result, the vestibular system may contribute differently to detecting slip or trip onset. For example, deviations in joint angles and joint torques from normal walking are noted to occur distally to proximally following a slip [18, 19], suggesting somatosensory information at the foot may play a large role in detection of slip onset. In contrast, deviations in joint angles and joint torques from normal walking tend to occur proximally than distally following a trip [20], suggesting there is potential for the vestibular system to contribute to detection of trip onset.

Previous studies have shown individuals with vestibular dysfunction can be 12-times more likely to fall [21], and the incidence of falls is greater for bilateral compared to unilateral vestibular dysfunction [22]. Such evidence supports the importance of the vestibular system in fall prevention. However, it is not known whether individuals with vestibular dysfunction are more susceptive to falls induced by slipping or tripping in particular. Determining whether head acceleration upon slipping and tripping differ significantly from head acceleration while walking would help determine whether the vestibular system could potentially contribute to detecting slip/trip onset as well as the subsequent balance recovery response. This information would help determine whether individuals with vestibular dysfunction are likely to be at a greater risk of falls due to slipping or tripping, and would inform the potential development of assistive devices providing augmented sensory feedback for vestibular dysfunction. Therefore, the purpose of this study was to investigate the effects of slipping and tripping on head acceleration while walking. It was hypothesized that (1) peak head acceleration after slipping and tripping would differ from those experienced while walking, and (2) deviations in head acceleration from walking would occur sooner after tripping compared to after slipping. The first hypothesis was based upon expectations that altered foot kinematics resulting from slipping or tripping would lead to changes in head acceleration compared to unperturbed walking. This second hypothesis was based upon expectations that the impulsive force applied to the swing foot when tripped would affect head acceleration earlier than the lack of friction force when slipped.

## Materials and Methods

Twelve young men completed this study (mean ± standard deviation, age: 20.9 ± 2.2 years, mass: 69.9 ± 4.4 kg, height: 177.8 ± 6.3 cm). Participants were recruited from the local university population using posted advertisements and email announcements, and were required to self-report no musculoskeletal, neurological, or balance disorders that influenced gait. This study was approved by the local Institutional Review Board, and written consent was obtained from all participants prior to participation.

Participants first performed several walking trials along a 10m level walkway covered in vinyl flooring. During these trials, participants were asked to walk naturally while looking straight ahead, and were told they would not be slipped or tripped. If mean gait speed during each trial (assessed using a motion capture system with marker on the inferior tip of the right scapula) was not between 1.45 to 1.60 m/s, participants were asked to increase or decrease their speed and repeat the trial. Speed was constrained to prevent large variations in speed from influencing head acceleration [23], and this speed range was selected as a purposeful (i.e. slightly hurried) walking speed for young adults [24, 25]. Participants performed 15–20 walking trials to familiarize themselves with the lab setting, feedback procedure, and gait speed, after which three trials were collected for analysis.

After walking trials were completed, participants were informed that at any point during the remainder of the session they may or may not be slipped or tripped as they walked down the walkway, and if so, they should attempt to maintain their balance and continue walking. To prevent auditory or visual cues of the slip or trip, noise protection earmuffs were worn, nature sounds were played, and the laboratory lighting was dimmed. Participants began each trial sitting on a stool at one end of the walkway with their back to the walkway. While sitting, they were given a set of letters, numbers, or symbols to memorize. The investigators then notified the participant to turn around and prepare to walk to the other end of the walkway. Once reaching the other end of the walkway, participants sat on another stool, attempted to write the memorized sequence, and then began to memorize a new sequence to remember during the next trial. This memorization task was an attempt to divert their attention away from a possible slip or trip. After repeating this for a minimum of 20 walking trials, six of the participants were unexpectedly slipped, while the other six were unexpectedly tripped, during a randomly selected trial. Each participant was slipped or tripped only once because previous research has shown that gait characteristics are altered following exposure to a slip or a trip [26–28]. Exposing a participant to both a slip or trip would significantly influence gait characteristics, and likely head acceleration, for the second perturbation. The right foot (preferred foot to kick a ball for all but one participant) was tripped or slipped. To elicit a slip, a foam paint roller was used to apply 50 ml of vegetable oil uniformly over a 90 x 90 cm area near the middle of the walkway while participants had their back to the walkway. Trips were induced in mid-to-late swing phase of gait using a manually actuated 7-cm-high obstacle embedded in the floor. All participants wore the same model of walking shoes in their requested size, and wore a safety harness attached to a track above the walkway to prevent impact with the floor in the event of an unsuccessful balance recovery.

Body segment positions and head acceleration were collected during all trials. Body segment positions were sampled at 200 Hz using a Vicon MX motion capture system (Vicon Motion Systems, Inc., Los Angeles, CA, USA), and reflective markers on the inferior tip of the right scapula, back of the head, temples, heels, lateral malleoli, and heads of fifth metatarsals. Tri-axial linear acceleration of the head was sampled at 800 Hz using a lightweight (55 g) six degree-of-freedom inertial measurement unit (IMU) (Memsense, LLC., Rapid City, SD, USA) attached to the forehead using double-sided tape and wrapped with elastic cohesive bandage (Co-flex, Andover Healthcare, Inc., Salisbury, MA, USA). Marker position and acceleration data were low-passed filtered at 5 and 20 Hz, respectively (fourth-order, zero-phase-shift Butterworth filter), prior to further processing.

Tri-axial linear acceleration of the right and left vestibular organs was calculated using methods similar to Rivera et al. [29]. Briefly, a head-fixed reference frame was first defined using three markers on the head (back of head, right temple, left temple). This reference frame had an origin at the right temple marker, a (+) x-axis defined by a unit vector pointing from the right temple marker to the left temple marker, a (+) z-axis defined by a unit vector perpendicular to the plane created by the back head marker, right temple marker, and left temple marker, and a (+) y-axis defined as perpendicular to the (+) x and (+) z-axis. Next, a pointer with three non-collinear markers was used to define landmarks on the head within this reference frame. These landmarks were used to define, as described below, the location of the vestibular organs, location of the IMU, orientation of the IMU, and orientation of another head-fixed reference frame that was aligned with the anatomical axes of the head (anatomical reference frame). These landmarks included the right and left anterior-superior point of the helix of the outer ear, right and left tragion, right and left mastoid process, right and left infraorbitale foramen, and entrance of the right and left ear canal. The location of the vestibular organs were defined to be at the intersection of a sagittal plane passing through at the mastoid process, a frontal plane passing through the infraorbitale foramen, and a transverse plane passing through the ear canal[30]. The anatomical reference frame had an origin at the head center of gravity (CG), and was defined by the anterior-posterior (AP), medial-lateral (ML) and vertical (VER) axes when in the anatomical position. The head CG was defined as the midpoint between the right and left anterior-superior point of the helix of the outer ear [31]. The (+) ML axis was defined as a unit vector pointing from the left to right tragion, the (+) VER axis was defined as a unit vector perpendicular to a plane created by the left tragion, right tragion, and average position of the left and right infraorbitale foramen (positive from head towards ground), and the (+) AP axis was defined as perpendicular to the (+) ML and (+) VER axis [32]. Acceleration during all trials was recorded within the IMU reference frame. Principles of relative acceleration from rigid body dynamics [33] were used to express this acceleration in the anatomical reference frame, and then to determine the acceleration of the vestibular organs. Head acceleration was calculated at the vestibular organs because it is at these locations that the body transduces accelerations into vestibular information.

To compare head acceleration upon slipping or tripping to those during walking, the maximum and minimum acceleration in each direction (AP, ML, and VER) were determined over intervals from 0-50ms, 50-100ms, 100-150ms, 150-200ms, 200-250ms, and 250-300ms following perturbation onset. The term *maximum acceleration* referred to the peak anterior, rightward, and superior acceleration for the AP, ML, and VER directions, respectively. Similarly, the term *minimum acceleration* referred to the peak posterior, leftward, and inferior acceleration for the AP, ML, and VER directions, respectively. Because differences between right and left vestibular organ acceleration were small, the larger magnitude acceleration between the right and left vestibular organ was used for each direction and time interval. For these calculations, perturbation onset during the walking trials was considered to be the instant of right foot heel contact for participants who were slipped, and the instant of minimum toe clearance of the right foot for participants who were tripped.

A two-way repeated measures analysis of variance on the ranks (due to non-normal distribution of residuals) for each dependent variable (peak acceleration in the anterior, posterior, rightward, leftward, superior, and inferior directions) was used to investigate the differences between conditions (slipping/tripping or walking) and time intervals (the six intervals listed above). A random effect for subject was included in the model to account for multiple measurements made during walking trials on each participant. In the event of a significant condition by time interval interaction, contrasts were performed within each time interval to investigate differences between slipping/tripping and walking. All statistical analyses were conducted using JMP Pro 11 (Cary, North Carolina, USA) with a significance level of *p*≤0.05.

## Results

**Fig 1:**
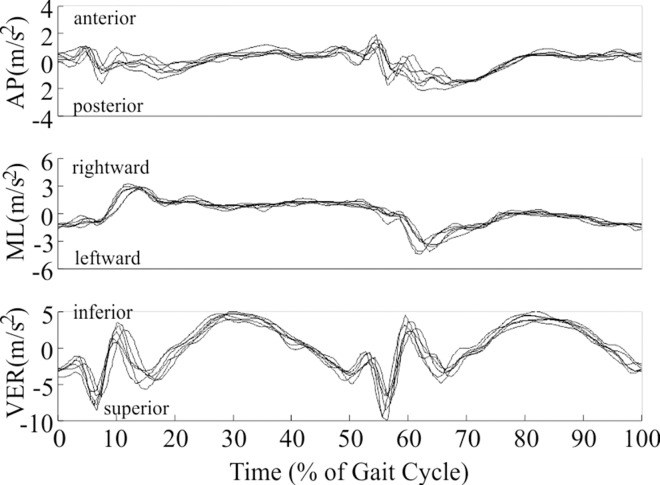
Acceleration (m/s^2^) of the right vestibular organ during walking for a representative participant. 0% and 100% of gait cycle both represent heel contact of the left foot, while 50% of gait cycle represents the approximate time of heel contact of the right foot. Six complete gait cycles are displayed.

**Fig 2:**
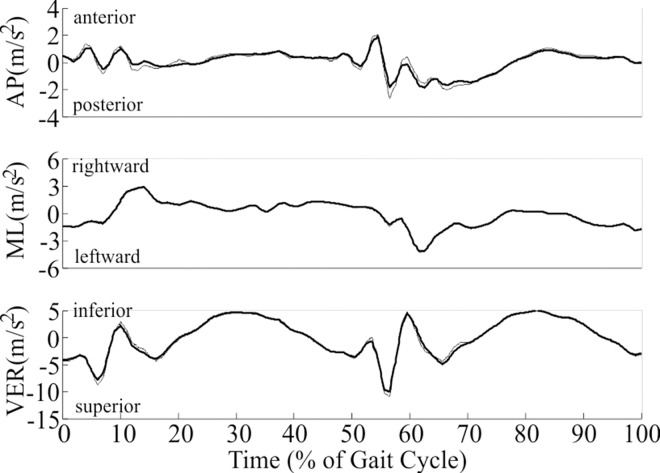
Accelerations (m/s^2^) of the right (thick solid line) and left (thin line) vestibular organs during a selected walking stride for a representative participant. This figure illustrates the subtle differences in accelerations measured between right and left vestibular organs.

**Fig 3:**
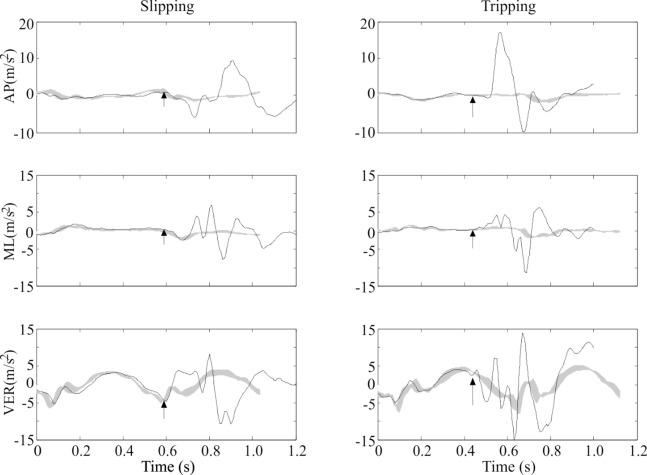
**Head acceleration when (a) slipping (black line) and walking (gray line), and (b) tripping (black line) and walking (gray line) for a representative participant.** The instant of slip and trip onset is denoted by an arrow. Head acceleration during walking is shown from left heel contact to left heel contact, and head acceleration during slipping and tripping are shown from heel contact of non-perturbed foot before perturbation (left for all participants) to subsequent heel contact of non-perturbed foot (left for all participants).

Head acceleration during walking showed a consistent pattern both within (Fig 1) and across all participants. Head acceleration across all participants ranged from 3.14 to -4.91 m/s^2^ in the AP direction (+ indicates anterior), 4.54 to -4.68 m/s^2^ in the ML direction (+ indicates rightward), and 6.25 to -10.98 m/s^2^ in the VER direction (+ indicates inferior). Only subtle differences in acceleration between left and right vestibular organs were measured (Fig 2) and attributed to rotations of the head and small bilateral discrepancies in the estimated location of the vestibular organs. Head acceleration after slipping (Fig 3A) and tripping (Fig 3B) deviated substantially from the pattern exhibited while walking, and exhibited greater inter-subject variation compared to walking. Peak head acceleration after tripping was higher after tripping than slipping, and deviations from walking occurred more quickly after tripping than slipping.

**Fig 4:**
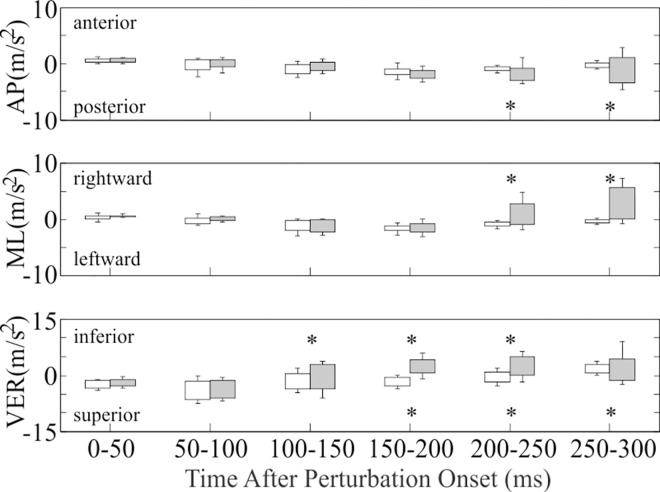
Peak head acceleration after slip onset (light grey) and heel contact during walking (white). The top of each column and the positive error bar indicates the median value and 75% percentile, respectively, in the positive direction (anterior, right, and inferior). The bottom of each column and the negative error bar indicates the median value and 75% percentile, respectively, in the negative direction (posterior, left, and superior). * denotes significant difference between slipping and walking within the time interval (*p*<0.05).

Head acceleration after slipping differed from walking in the AP, ML, and VER directions (Table 1; Fig 4). AP acceleration after slipping differed from walking in that the median peak posterior acceleration was 1.64–2.68 m/s^2^ higher after slipping during the 200-250ms (*t* = 4.37; *df* = 246 for this and all subsequently reported contrasts on slipping; *p*<0.001) and 250-300ms (*t* = 6.26; *p*<0.001) time intervals. ML acceleration after slipping differed from walking in that the median peak rightward acceleration was 3.18–5.68 m/s^2^ higher after slipping during the 200-250ms (*t* = 4.99, *p*<0.001) and 250-300ms (*t* = 4.21; *p*<0.001) time intervals. VER acceleration after slipping also differed from walking in that the median peak inferior acceleration was 2.39–4.68 m/s^2^ higher after slipping during the 100-150ms (*t* = 3.72; *p*<0.001), 150-200ms (*t* = 7.02; *p*<0.001), and 200-250ms (*t* = 5.23; *p*<0.001) time intervals. Additionally, the median peak superior acceleration was 2.10 m/s^2^ higher after slipping during the 250-300ms (*t* = 4.31; *p*<0.001) time interval, but was 1.89–3.63 m/s^2^ lower after slipping during the 150-200ms (*t* = 4.72; *p*<0.001) and 200-250ms (*t* = 2.20; *p* = 0.029) time intervals. Most often, the median peak superior acceleration during the 150-200ms and 200-250ms time intervals was actually the minimum inferior acceleration due to the absence of superiorly-directed acceleration during these time intervals.

**Table 1:** Peak head acceleration after slip onset and heel contact during walking.

		0–50 ms	50–100 ms	100–150 ms	150–200 ms	200–250 ms	250–300 ms
**Anterior**	**Walk**	0.83 ± 0.62	0.56 ± 0.64	-0.33 ± 0.93	-0.76 ± 0.87	-0.59 ± 0.48	0.11 ± 0.54
	**Slip**	0.80 ± 0.37	0.65 ± 0.50	0.10 ± 0.79	-1.07 ± 0.56	-0.28 ± 1.51	1.21 ± 1.97
**Posterior**	**Walk**	0.07 ± 0.70	-1.13 ± 1.07	-1.74 ± 0.88	-1.81 ± 0.91	-1.42 ± 0.55	-0.87 ± 0.43
	**Slip**	0.12 ± 0.39	-0.64 ± 1.11	-1.43 ± 0.62	-2.50 ± 1.01	-3.73 ± 1.69 *	-5.05 ± 4.38 *
**Rightward**	**Walk**	0.77 ± 0.78	0.49 ± 0.62	-0.37 ± 0.86	-1.00 ± 0.99	-0.39 ± 0.67	-0.01 ± 0.37
	**Slip**	0.71 ± 0.59	0.46 ± 0.25	-0.39 ± 0.73	-0.39 ± 1.11	2.71 ± 2.63 *	4.65 ± 3.04 *
**Leftward**	**Walk**	0.12 ± 0.72	-0.81 ± 0.63	-1.98 ± 1.26	-1.88 ± 1.23	-1.18 ± 0.86	-0.57 ± 0.51
	**Slip**	0.27 ± 0.39	-0.51 ± 0.69	-2.36 ± 1.09	-2.06 ± 1.03	-0.47 ± 1.14	-0.84 ± 2.38
**Inferior**	**Walk**	-1.67 ± 0.91	-1.15 ± 1.66	0.64 ± 1.50	-0.50 ± 1.18	0.96 ± 1.16	2.92 ± 0.99
	**Slip**	-1.35 ± 1.14	-1.16 ± 0.86	2.63 ± 1.36 *	4.16 ± 2.68 *	5.59 ± 2.57 *	5.53 ± 3.24
**Superior**	**Walk**	-3.79 ± 1.39	-6.46 ± 1.39	-3.59 ± 1.61	-2.85 ± 1.59	-1.50 ± 1.14	0.74 ± 1.13
	**Slip**	-3.21 ± 1.30	-6.09 ± 0.98	-3.86 ± 2.67	0.47 ± 2.36 *	0.30 ± 1.90 *	-2.02 ± 3.06 *

Values are given as: mean ± standard deviation in m/s^2^.

* on slipping values denotes significant difference from walking within the time interval (*p*<0.05).

**Fig 5:**
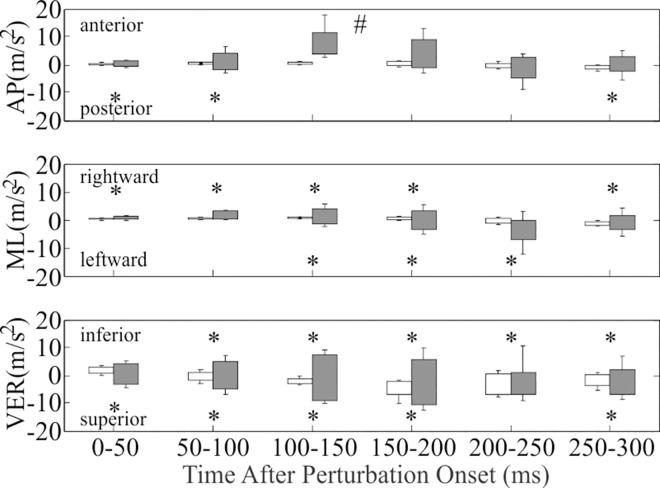
Peak head acceleration after trip onset (dark grey) and mid-swing during walking (white). The top of each column and positive error bar indicates the median value and 75% percentile, respectively, in the positive direction (anterior, right, and inferior). The bottom of each column and negative error bar indicates the median value and 75% percentile, respectively, in the negative direction (posterior, left, and superior). * denotes significant difference between tripping and walking within the time interval (*p*<0.05). # denotes a significant main effect of condition across all time intervals (*p*<0.001).

Head acceleration after tripping differed from walking in the AP, ML, and VER directions (Table 2; Fig 5). AP acceleration after tripping differed from walking in that the median peak anterior acceleration was 0.88–10.65 m/s^2^ higher after tripping over all time intervals (*F* = 154.4; *df* = 235 for this and all subsequently reported contrasts on tripping; *p*<0.001). In addition, the median peak posterior acceleration was 0.50–1.86 m/s^2^ higher after tripping over the 0-50ms (*t* = 2.57; *p* = 0.011), 50-100ms (*t* = 5.34; *p*<0.001), and 250-300ms (*t* = 2.24; *p* = 0.026) time intervals. ML acceleration after tripping differed from walking in that the median peak rightward acceleration was 0.74–3.20 m/s^2^ higher after tripping over all time intervals (*t* > 2.39; *p*<0.018), excluding 200-250ms (*t* = 1.08; *p* = 0.283). In addition, the median peak leftward acceleration was 2.02–5.90 m/s^2^ higher after tripping over the 100-150ms (*t* = 6.91; *p*<0.001), 150-200ms (*t* = 6.26; *p*<0.001) and 200-250ms (*t* = 2.91; *p* = 0.004) time intervals. VER acceleration after tripping also differed from walking in that the median peak inferior acceleration was 0.70–8.85 m/s^2^ higher after tripping over all time intervals (*t* > 2.56; *p*<0.012), excluding 0-50ms (*t* = 1.48; *p* = 0.141). In addition, the median peak superior acceleration was 3.12–6.14 m/s^2^ higher after tripping over all time intervals (*t* > 3.47; *p*<0.001), excluding 200-250ms (*t* = 0.90; *p* = 0.370).

**Table 2:** Peak head acceleration after trip onset and heel contact during walking.

		0–50 ms	50–100 ms	100–150 ms	150–200 ms	200–250 ms	250–300 ms
**Anterior**	**Walk**	0.58 ± 0.33	0.76 ± 0.29	0.90 ± 0.46	0.99 ± 0.58	0.41 ± 0.86	-0.42 ± 1.05
	**Trip** ^**#**^	1.27 ± 0.62	4.34 ± 2.44	12.91 ± 4.25	10.10 ± 4.10	2.56 ± 1.60	2.83 ± 2.68
**Posterior**	**Walk**	0.26 ± 0.30	0.27 ± 0.26	0.32 ± 0.48	-0.29 ± 0.97	-1.02 ± 0.99	-1.62 ± 1.05
	**Trip**	-0.21 ± 0.42 *	-1.45 ± 1.27 *	3.27 ± 2.97	-0.40 ± 2.46	-4.64 ± 4.08	-2.27 ± 3.72 *
**Rightward**	**Walk**	0.75 ± 0.32	0.93 ± 0.28	1.04 ± 0.28	1.01 ± 0.38	0.73 ± 0.73	-0.80 ± 0.94
	**Trip**	1.37 ± 0.47 *	3.07 ± 0.93 *	4.22 ± 2.60 *	3.63 ± 2.82 *	0.61 ± 2.20	1.82 ± 3.03 *
**Leftward**	**Walk**	0.35 ± 0.33	0.56 ± 0.28	0.60 ± 0.33	-0.03 ± 0.79	-1.35 ± 1.24	-2.16 ± 0.86
	**Trip**	0.19 ± 0.68	0.32 ± 0.46	-2.22 ± 2.61 *	-3.49 ± 2.32 *	-6.32 ± 4.19 *	-4.70 ± 4.51
**Inferior**	**Walk**	2.81 ± 1.05	1.37 ± 1.12	-0.86 ± 1.09	-1.71 ± 1.44	0.33 ± 2.04	0.45 ± 1.15
	**Trip**	4.40 ± 1.19	5.25 ± 2.05 *	7.18 ± 2.91 *	5.74 ± 4.13 *	4.27 ± 6.08 *	3.11 ± 3.53 *
**Superior**	**Walk**	1.26 ± 1.09	-1.27 ± 1.37	-2.70 ± 1.09	-6.07 ± 2.49	-6.19 ± 1.88	-3.31 ± 1.88
	**Trip**	-2.62 ± 1.49 *	-5.60 ± 2.82 *	-9.41 ± 2.30 *	-10.70 ± 2.94 *	-7.35 ± 3.37	-7.08 ± 2.83 *

Values are given as: mean ± standard deviation in m/s^2^.

* on tripping values denotes significant difference from walking within the time interval (*p*<0.05).

^#^ denotes a significant difference between tripping and walking for all time intervals (*p*<0.05).

## Discussion

The purpose of this study was to investigate the effects of slipping and tripping on head acceleration while walking. Our first hypothesis was that peak head acceleration after slipping and tripping would exceed those experienced while walking. This hypothesis was supported because peak accelerations were up to 4.68 m/s^2^ higher after slipping, and up to 10.64 m/s^2^ higher after tripping, compared to walking. Our second hypothesis was that differences in head acceleration from walking would occur sooner after tripping compared to after slipping. This hypothesis was supported because differences from walking were found 0–50 ms after trip onset, but not until 100–150 ms after slip onset. The initial deviation following a slip involved the head accelerating more inferiorly compared to walking, and continued until 250–300 ms after slip onset when the head accelerated more superiorly compared to walking. Head acceleration was also more rightward (toward the foot that slipped forward) 200–300 ms after slip onset, and more posterior 200–300 ms after slip onset, compared to walking. These accelerations were consistent with head movement during a backward fall to the same side as the slipping foot, and likely result from less than expected support from the slipping leg. The initial deviation following a trip involved the head accelerating more anteriorly and posteriorly, rightwardly, and superiorly compared to walking. After this 0–50 ms time interval, numerous deviations in head acceleration from walking persisted throughout the initial 300 ms after tripping that was investigated here. These accelerations were generally consistent with head movement during a forward fall to the same side as the tripped foot. However, increases in median peak head acceleration in all six directions indicated head acceleration after tripping was more complex than after slipping.

Head accelerations while walking found here were similar to previous studies[11, 23, 25, 34–36]. Root-mean-square (RMS) acceleration calculated over the entire gait cycle in the current study (compared to the range of mean values reported elsewhere) was 0.10 ± 0.04 g (0.10 to 0.11 g) in the AP direction, 0.10 ± 0.03 g (0.08 to 0.17 g) in the ML direction, and 0.29 ± 0.04 g (0.17 to 0.21 g) in the VER direction[11, 23, 25, 34–36]. The higher RMS acceleration in the VER direction of the current study was likely due to a faster walking speed (1.45–1.60 m/s) compared to prior studies (1.2–1.3 m/s). Peak head acceleration and RMS acceleration tends to increase with gait speed, and more so in the VER direction compared to AP and ML directions[23, 25]. Other small discrepancies between studies were expected due to prior studies estimating head acceleration at the posterior aspect[36] and vertex[11, 23, 25, 34] of the head.

Head acceleration differences must be of sufficient magnitude to be detected by the vestibular system in order to contribute to onset detection and balance recovery response after slipping and tripping. The estimated vestibular thresholds for the detection of linear acceleration from a static initial position are 0.063 m/s^2^ in AP direction, 0.057 m/s^2^ in ML direction, and 0.154 m/s^2^ in VER direction[37]. Median/mean head acceleration while walking reported here and elsewhere [11, 23, 25, 34–36] exceeded these thresholds at heel contact when slips commonly occur (0.18 to 0.80 m/s^2^ in AP direction, 0.10 to 0.66 m/s^2^ in ML direction, -3.56 to -1.42 m/s^2^ in VER direction), and at mid-swing when trips commonly occur (0.21 to 0.59 m/s^2^ in AP direction, 0.27 to 0.69 m/s^2^ in ML direction, 1.14 to 3.11 m/s^2^ in VER direction). In addition, the difference in median peak acceleration between slipping and walking, as well as between tripping and walking, at these critical times within the gait cycle also exceeded these thresholds in each direction during all time intervals (although not all differences reach statistical significance). This suggests head accelerations were of sufficient magnitude to help detect trip onset as well as the balance recovery response after slipping and tripping. However, it is unclear if the static thresholds reported above can be generalized to the dynamic situation of walking when the body does not start from a static position.

In addition to being of sufficient magnitude, deviations in head acceleration must occur early enough to contribute to onset detection and to contribute to the balance recovery response after slipping and tripping. Previous studies have shown lower limb muscle latency times of 90–300 ms after the onset of a slip[38–40], with an initial knee flexor response in the slipping leg, followed by a knee extensor response in the slipping leg[38, 41, 42]. This initial response included activity of the bicep femoris and tibialis anterior muscles, which typically have the shortest muscle latency times after slipping, ranging from 90 to 160 ms[38, 40, 42]. Because differences in head acceleration between slipping and walking reported here were not found until 100–150 ms after slip onset, these findings suggest that the vestibular system was not likely involved in detecting slip onset. Furthermore, previous research has demonstrated that following a slip, vertical ground reaction force deviates from normal walking approximately 58 ms after heel contact followed by deviations in knee angle and angular velocity at 116 and 111ms after heel contact, respectively [19]. Combined, these results and ours would suggest that somatosensory information from the foot might be the primary mechanism towards detection of perturbation onset and initiation of the balance recovery response. This is further supported by Choi et al. [18], who demonstrated that peak plantar pressures in the forefoot occur earlier while peak plantar pressures in the heel occur later during slipping compared to walking. This also suggests that somatosensory information is detected by and used to alter loading at the foot for recovery. However, this does not preclude a vestibular contribution later in the balance recovery response when differences from walking were found. It is also important to note that while some previous studies used similar methods to induce a slip[38, 41], others used a sliding platform[39, 40, 42], and differences in slipping foot kinematics between these two methods [43] could contribute to differences in muscle latency times.

In regards to tripping, previous studies have shown muscle latency times of 55–150 ms following trip onset[20, 44]. This initial response included activity of the bicep femoris, which typically had the shortest muscle latency times, to initiate a knee flexor response in the swing leg to clear the obstacle and an hip extensor response in the stance limb to arrest forward momentum and increase the height of the body center of mass. Furthermore, following a trip, deviations in joint angles and torques from normal walking occur first at the hip approximately 125–150 ms after trip onset, then knee and ankle approximately 160–260 ms after trip onset [20]. Together, these and our findings suggest that deviations in head accelerations occur prior to initiation of the balance recovery response and deviation of lower limb joints from normal walking, and it is plausible for the vestibular system to help detect trip onset. As after slipping, later differences from walking may have also contributed to the balance recovery response.

Based upon the results presented here, vestibular dysfunction is not expected to affect the detection of slip onset, but may affect the detection of trip onset. It is also possible for vestibular dysfunction to have an adverse effect on later aspects of the balance recovery response after slipping or tripping. However, sensory re-weighing is also possible to accommodate for dysfunction. The results presented here may also provide guidance in the development of sensory augmentation devices to detect losses of balance due to slipping and tripping.

Several limitations to this study warrant mention. First, we only assessed linear acceleration of the head. The vestibular system also provides feedback on head orientation and angular velocity, and these may provide additional information that contribute to the detection of slip/trip onset, and the balance recovery response. Second, while it is known that the vestibular system is sensitive to acceleration, it is not known how acceleration information is processed and utilized for detecting the onset of a slip or a trip. As such, we are only able to infer a contribution of the vestibular system to the actual detection of a slip or trip, as well as the subsequent motor response. Third, anticipation-related effects may have existed when slipping or tripping. However, efforts were made to minimize any such effects using a memorization task, by requiring participants to complete at least 20 trials before being slipped/tripped, and by slipping or tripping participants unexpectedly. Fourth, testing was performed with young adults. Generalizing these results to other populations should be done with caution. Finally, due to the small sample size, additional follow up studies are needed to validate these results and support generalization to larger populations.

## Conclusions

In conclusion, head acceleration after slipping and tripping exceeded those while walking. The temporal characteristics of head acceleration support a possible contribution of the vestibular system to detecting trip onset, but not slip onset. Head acceleration after slipping and tripping also appeared to be sufficiently large to contribute to the balance recovery response.

## Supporting Information

S1 File Participant Anthropometry and Trial Data. This file contains a sheet with participant anthropometry, variable definitions, and time series data for each trial for each participant.
(XLSX)
